# Hydrophobic hydration of poly-N-isopropyl acrylamide: a matter of the mean energetic state of water

**DOI:** 10.1038/srep04377

**Published:** 2014-03-14

**Authors:** I. Bischofberger, D. C. E. Calzolari, P. De Los Rios, I. Jelezarov, V. Trappe

**Affiliations:** 1 University of Fribourg, Department of Physics, CH-1700 Fribourg; 2 Ecole Polytechnique Fédérale de Lausanne, Laboratory of Statistical Biophysics, CH-1015 Lausanne; 3 University of Zürich, Department of Biochemistry, CH-8057 Zürich; 4 Current address: University of Chicago, Department of Physics, Chicago, IL 60637.

## Abstract

The enthalpically favoured hydration of hydrophobic entities, termed hydrophobic hydration, impacts the phase behaviour of numerous amphiphiles in water. Here, we show experimental evidence that hydrophobic hydration is strongly determined by the mean energetics of the aqueous medium. We investigate the aggregation and collapse of an amphiphilic polymer, poly-N-isopropyl acrylamide (PNiPAM), in aqueous solutions containing small amounts of alcohol and find that the thermodynamic characteristics defining the phase transitions of PNiPAM evolve relative to the solvent composition at which the excess mixing enthalpy of the water/alcohol mixtures becomes minimal. Such correlation between solvent energetics and solution thermodynamics extends to other mixtures containing neutral organic solutes that are considered as kosmotropes to induce a strengthening of the hydrogen bonded water network. This denotes the energetics of water as a key parameter controlling the phase behaviour of PNiPAM and identifies the excess mixing enthalpy of water/kosmotrope mixtures as a gauge of the kosmotropic effect on hydrophobic assemblies.

Water is without doubt the most important solvent on earth, both in respect to biologically as well as technologically relevant systems^1^,^2^. Besides being the most abundant liquid, the importance of water as a solvent is largely due to its capacity to solvate polar as well as apolar entities. The hydration of the latter is termed hydrophobic hydration; a hydration process that is enthalpically favoured at the cost of being unfavourable to entropy^3^,^4^. The thermodynamics of this phenomenon can be understood as a pure solvent problem, where the inclusion of a hydrophobe into water leads to the formation of a hydration shell, which is enthalpically favourable to water, as the water molecules in the hydration shell form stronger and longer-lived hydrogen bonds than in the bulk^2^,^5^,^6^,^7^. As a result of this, the phase behaviour of amphiphiles and hydrophobes in water exhibits a temperature dependence that is reverse to that normally observed. Amphiphiles and hydrophobes dissolve in cold water, but aggregate and phase separate at higher temperatures owing to the entropy gain obtained by releasing the water molecules from the hydration shell^8^,^9^,^10^. Such water mediated attraction among hydrophobes governs the phase behaviour and self-assembly of numerous amphiphilic molecules in aqueous media; examples are protein folding, the assembly of lipids in membranes and the micelle formation of surfactants^11^,^12^,^13^. Despite the undisputable importance of such processes a general consensus regarding the origin of hydrophobic hydration and hydrophobic interactions has to date not been reached^3^,^4^. The main reason for this is that hydrophobicity is a multiple facetted problem that depends on the shape and size of the hydrophobic entity as well as on temperature^4^. This complexity has led to extensive investigations and controversial discussions that to some extent prevent the development of simple concepts and guidelines for the wide community of scientists working in other fields like for instance materials and pharmaceutics, where hydrophobic assemblies or the thermosensitivity of amphiphilic polymers are of great interest^14^,^15^,^16^,^17^,^18^.

In this work, we show experimental evidence that hydrophobic hydration is strongly determined by the mean energetic state of water that is tuned by the addition of an organic solute classified as kosmotrope. The presence of small amounts of such solute in water is generally believed to strengthen the hydrogen-bonded network of water, which is known as the kosmotropic effect^19^,^20^,^21^. We show that when hydrophobic hydration is the prevailing contribution controlling the phase behaviour of an amphiphilic polymer, such as poly-N-isopropyl acrylamide (PNiPAM), the impact of a given kosmotrope on the phase behaviour is directly correlated to the energetics of the solvent; thermodynamic characteristics defining the phase transition of PNiPAM evolve relative to the solvent composition at which the excess mixing enthalpy of the water/kosmotrope mixtures becomes minimal. Regarding the fundamental aspects of hydrophobic hydration this correlation exposes the coarse grained nature of hydrophobic hydration that strongly depends on the mean energetics of the aqueous medium, in support of theoretical approaches such as the two-state model that describe hydrophobic hydration as resulting from the energy difference between bulk and shell water^8^,^20^,^22^,^23^. Regarding the tunability of hydrophobic assemblies this correlation provides a valuable guideline on how to predict the impact of the kosmotropic effect when using different types of kosmotropic organic solutes, without the need of developing a full thermodynamic model, which can be very complex in such multi-component systems.

## Results

### Molecular weight and concentration independence of the phase behaviour of PNiPAM in water/alcohol mixtures

We investigate the solvation behaviour of poly-N-isopropyl acrylamide (PNiPAM) in water and water/alcohol mixtures aiming in a first step to rationalize a number of previous observations. PNiPAM is a neutral amphiphilic polymer that dissolves in cold water but exhibits a coil-to-globule transition and precipitates out of solution as the temperature is increased beyond a lower critical solution temperature (LCST)^14^. Several investigations have shown that the LCST of PNiPAM in water is almost independent of PNiPAM molecular weight and concentration^24^,^25^, yet others have shown that the LCST significantly decreases upon addition of small amounts of alcohol^26^,^27^,^28^. To fully assess these features we here explore how the critical solution temperatures depend on PNiPAM molecular weight and concentration at various alcohol molar fractions *X*.

**Figure 1:**
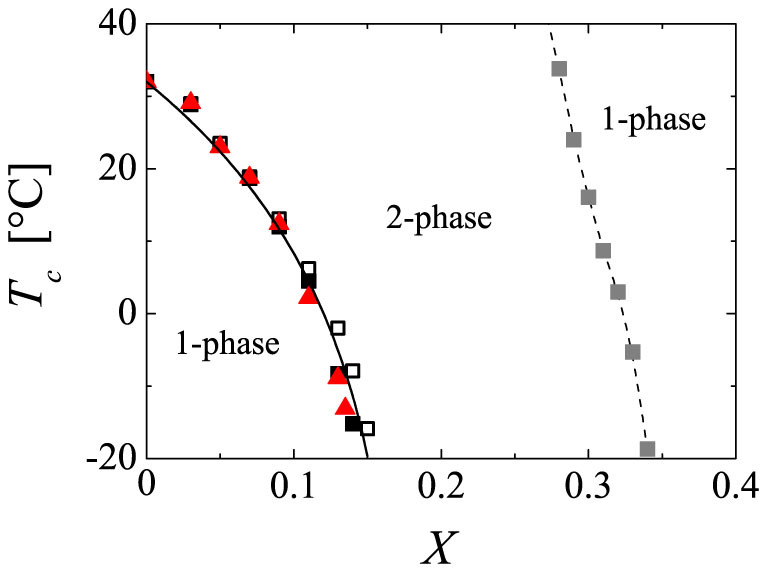
Dependence of critical solution temperature *T_c_* on ethanol molar fraction *X*. The data are obtained for a PNiPAM system with *M_v_* = 39 000 g/mol at concentrations of *c* = 10^−2^ g/ml (full squares) and *c* = 2**·**10^−3^ g/ml (open squares) and for a PNiPAM system with *M_v_* = 465 500 g/mol at a concentration of *c* = 10^−2^ g/ml (full triangles). The LCST exhibits a strong dependence on *X*, while it is essentially independent of the molecular weight and concentration of PNiPAM. At higher ethanol content there is a second boundary (grey squares), characterized by a UCST, beyond which PNiPAM is soluble in the entire range of accessible temperatures. The indicated boundary is that measured for PNiPAM with *M_v_* = 39 000 g/mol at a concentration of *c* = 10^−2^ g/ml; note, however, that the exact position of this boundary depends on both molecular weight and concentration. The continuous and dashed lines through the data are guides to the eye.

As a typical example for PNiPAM in water/alcohol mixtures we investigate the phase behaviour of PNiPAM in water/ethanol mixtures in more detail, but let us note that tests with other types of alcohols confirmed the general conclusions that are drawn from this investigation. The critical solution temperatures *T_c_* obtained in cloud point measurements (see methods) are shown in Fig. 1. PNiPAM exhibits in water/ethanol mixtures the somewhat unusual phenomenon of co-nonsolvency, which denotes that a system may be perfectly soluble in two different solvents, but not in certain mixtures of both^26^,^28^,^29^,^30^. At low *X* PNiPAM is fully soluble at low temperatures, but precipitates out of solution as the temperature is increased beyond the LCST. At intermediate *X* PNiPAM is insoluble within the temperature range of −20°C to 60°C accessible in our experiments, while being fully soluble within this temperature range at higher *X*. The boundary between the two-phase and one-phase regime observed at higher *X* is characterized by an upper critical solution temperature (UCST) that depends on both molecular weight and concentration of PNiPAM; as an example we show in Fig. 1 the UCST boundary obtained for a PNiPAM system with a viscosity averaged molecular weight of *M_v_* = 39 000 g/mol and a concentration of *c* = 10^−2^ g/ml.

The focus of this paper, however, is set on the boundary found in the lower range of *X*, where the transition from a one-phase to a two-phase regime is characterized by a LCST, which is a decreasing function of *X*. In this range of *X* the most striking feature in the phase behaviour of PNiPAM is that small variations in the alcohol content strongly impact the LCST, while a variation of the molecular weight or concentration of PNiPAM does not lead to a significant change of the LCST. Indeed, varying the molecular weight by more than a factor of 10 or varying the concentration by a factor of 5 leads to the same generic dependence of *T_c_* on *X*; this is shown in Fig. 1, where we display the *X*-dependence of the LCST obtained for solutions of PNiPAM systems with respectively *M_v_* = 39 000 g/mol and *M_v_* = 465 500 g/mol at a concentration of *c* = 10^−2^ g/ml, together with that obtained for the PNiPAM system with *M_v_* = 39 000 g/mol at a concentration of *c* = 2**·**10^−3^ g/ml. Consistent with the molecular weight and concentration independence of the LCST, the coil-to-globule transition and the onset to aggregated, phase separated states is found to coincide in the low range of *X*^28^,^29^,^31^, as this is the case for PNiPAM in pure water^24^,^32^. This shows that increasing *X* solely leads to a decrease of the absolute magnitude of the LCST; the characteristics of the phase behaviour observed in pure water remain the same for PNiPAM in water/ethanol mixtures with *X* < 0.15.

These characteristics, however, are in stark contrast to those expected for classical polymer solutions. There we expect the critical temperatures at which the system starts to phase separate or where a single polymer chain exhibits a coil-to-globule transition to depend on the degree polymerization, i.e. the molecular weight, of the polymer^33^. Fundamentally, this is owing to the fact that the monomeric units, of which the polymer is composed, are not free to explore all possible configurations as they are attached to each other. The larger the constraints set by the degree of polymerization the smaller the number of possible configurations and thus the lower the entropy. This impacts both the mixing entropy relevant to phase separation and the polymer configurational entropy relevant to the coil-to-globule transition, such that the transition temperatures of both transitions are generally functions of the polymer molecular weight^33^. Moreover, as the mixing entropy also depends on the concentration, the transition temperatures for phase separation normally depend on concentration^33^,^34^. This also entails that the coil-to-globule transition temperature, which is concentration independent, does not necessarily correspond to the transition temperatures denoting the onset to phase separation^34^.

In regard to the classical phase behaviour of polymer solutions the independence of the phase behaviour of PNiPAM on molecular weight and concentration is thus rather unusual. It effectively shows that the entropic contributions of the polymer are largely irrelevant for the phase behaviour of PNiPAM, which in turn indicates that other parameters must be governing the phase transitions of PNiPAM in aqueous media. The fact that the addition of small amounts of alcohol leads to a significant decrease of the LCST, while the LCST remains almost constant when varying the molecular weight or concentration of PNiPAM, suggests that the solvent must play a determining role. Indeed, in this work we show experimental evidence that it is the energetic state of the aqueous medium that predominantly governs the phase behaviour of PNiPAM and that this energetic state is tuned by the presence of small amounts of alcohol.

### Correlations between solvent enthalpy and lower critical solution temperature of PNiPAM in water/alcohol mixtures

**Figure 2:**
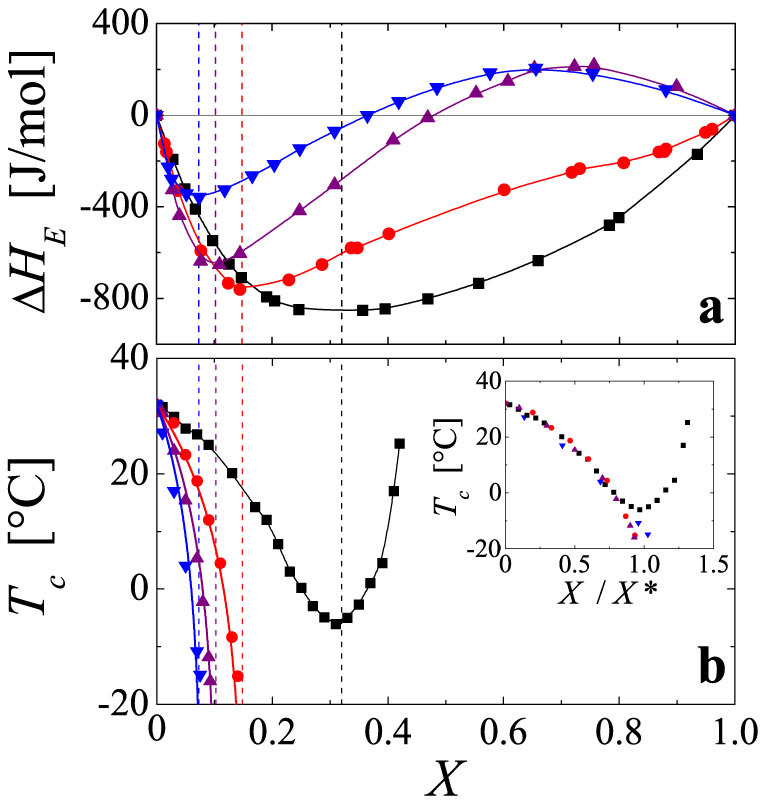
Thermodynamic properties of water/alcohol mixtures in comparison to those of PNiPAM in water/alcohol mixtures. Water/methanol (squares), water/ethanol (circles), water/iso-propanol (triangles up) and water/propanol (triangles down). (a) Data obtained by Lama and Lu^35^. Excess enthalpy of mixing Δ*H_E_*, resulting from mixing alcohols into water, as a function of alcohol molar fraction *X* as measured at 25°C. The continuous lines are guides to the eye. (b) Dependence of the lower critical solution temperature *T_c_* of PNiPAM on *X*. The experiments have been performed with the PNiPAM system with *M_v_* = 39 000 g/mol at a concentration of *c* = 10^−2^ g/ml. The continuous lines are guides to the eye. As denoted by the vertical dotted lines, the compositions at which the LCST become minimal, X*(*T_c_*), correspond to those where the excess enthalpies of mixing exhibit a minimum, X*(*H_E_*). Inset: Normalization of *X* with X*(Δ*H_E_*) leads to an almost perfect collapse of the critical temperatures obtained for the different water/alcohol mixtures.

As shown in Fig. 2(a), the excess enthalpy of mixing Δ*H_E_*, resulting from mixing alcohols into water, exhibits a minimum at a molar fraction X*(Δ*H_E_*), which sensitively depends on the type of alcohol used; it increases from propanol, iso-propanol, ethanol to methanol^35^. Strikingly, the exact same trend is also observed in the molar composition X*(*T_c_*) denoting the composition where the LCST exhibits a minimum for the PNiPAM solutions in water/methanol and appears to diverge to minus infinity for the PNiPAM solutions in mixtures of water with higher order alcohols, as shown in Fig. 2(b). In fact, X*(*T_c_*) almost exactly coincides with the minima of the excess enthalpy X*(Δ*H_E_*), as indicated by the vertical lines in Fig. 2. Moreover, a simple normalization of *X* with X*(Δ*H_E_*) leads to a nearly perfect collapse of the critical solution temperatures obtained for the different water/alcohol mixtures at low *X*, as shown in the inset of Fig. 2(b). This clearly shows that minimizing the solvent enthalpy is unfavourable to the hydration of PNiPAM. Moreover, this correlation denotes X*(Δ*H_E_*) as a gauge for the impact of a given alcohol on the LCST of PNiPAM; the LCST develops relative to X*(Δ*H_E_*), which is a function of the size and geometry of the alcohol used. Let us note that the absolute magnitude of Δ*H_E_* is a function of temperature; the *X*-dependence shown in Fig. 2(a) is that obtained at *T* = 25°C by Lama and Lu^35^. However, even though Δ*H_E_* is temperature dependent, for the temperature range of *T* ≤ 33°C relevant to the LCST-behaviour of PNiPAM the position of the minima does not depend on *T*, such that X*(Δ*H_E_*) can indeed be considered as unique for a given water/alcohol mixture^35^,^36^,^37^,^38^.

The direct correspondence of X*(*T_c_*) and X*(Δ*H_E_*) is not exclusively limited to water/alcohol mixtures, but also applies to mixtures of water with other organic solutes. In Table 1 we report a collection of data for X*(*T_c_*), X*(Δ*H_E_*) and the magnitude of Δ*H_E_* at X* obtained from literature^26^,^29^,^35^,^39^,^40^,^41^,^42^. In addition to the alcohols under consideration, we find that for acetone, dioxane and tetrahydrofuran (THF), X*(*T_c_*) = X*(Δ*H_E_*) within error bars, while X*(*T_c_*) and X*(Δ*H_E_*) are uncorrelated for mixtures of water with dimethylformamide (DMF) and dimethylsulfoxide (DMSO). It is significant that all solutes where X*(*T_c_*) = X*(Δ*H_E_*) are classified as kosmotropes^26^,^43^,^44^,^45^,^46^, while the solutes where X*(*T_c_*) ≠ X*(Δ*H_E_*) are classified as chaotropes^26^,^47^,^48^,^49^. As introduced above, kosmotropes are generally presumed to lead to a strengthening of the hydrogen bonds among water molecules without disrupting the water network^19^,^20^,^26^, while the addition of chaotropes is thought to disrupt the water network^26^,^48^,^49^. However, such definition is somewhat ambiguous, as it is not based on a well-defined measure of the water state or the water-solute interactions. Indeed, the classification into kosmotropes and chaotropes comprises a variety of different classes of solutes, ranging from inorganic salts to neutral organic molecules^21^,^48^,^50^,^51^. Considering the significantly different interactions between water and respectively inorganic ions and neutral organic molecules^21^,^48^,^50^,^51^,^52^, we expect that the molecular origin of the kosmotropic and chaotropic effects will depend on the type of solute used. For the kosmotropic neutral organic solutes listed in Table 1 there is evidence that the kosmotropic effect is related to the fact that these solutes do not form strong direct hydrogen bonds with water, inducing instead a structuring of water via the hydrophobic hydration of their hydrophobic groups^44^,^46^,^52^,^53^,^54^,^55^. By contrast, DMSO and DMF are known to form direct hydrogen bonds with water that are stronger than those among water molecules, which leads to the disruption of the water network^47^,^49^,^56^. We can presume that the decrease in the mixing enthalpy observed for the water/kosmotrope mixtures in the low *X*-range is mostly due to the hydrophobic hydration that induces the kosmotropic effect. The composition at which Δ*H_E_* becomes minimal then effectively defines the upper limit at which the addition of a kosmotrope to water mainly leads to a strengthening of the hydrogen bonds among water molecules. By contrast, for the water/chaotrope mixtures the formation of strong direct hydrogen bonds between the chaotrope and water are likely dominating the mixing enthalpy. Evidence for this is found when considering the magnitude of the excess enthalpy of mixing at X* that are listed in Table 1; Δ*H_E_*(X*) is almost a factor of three higher for the chaotropes than for the kosmotropes. It is thus reasonable to assume that Δ*H_E_* is not a direct measure of the strength of the hydrogen-bonded water network for the water/chaotrope mixtures, while this is case for the water/kosmotrope mixtures.

The fact that for the water/kosmotrope mixtures the solvation properties of PNiPAM are directly correlated to the enthalpy of the solvent mixture itself strongly suggests that the strength of the hydrogen bonds among water molecules sets the phase behaviour of PNiPAM. This in turn indicates that hydrophobic hydration and interactions are key contributions to the transitions between fully soluble and aggregated or collapsed states of PNiPAM. Beyond the molecular detailed considerations of the effect of kosmotropes on the water structure, we can consider the presence of kosmotropes to just lead to a decrease in the bulk water enthalpy. As the enthalpy of the bulk water decreases the gain in enthalpy for water to form a hydration shell around the hydrophobic groups of PNiPAM decreases, consequently the LCST decreases and eventually drops to a minimum or completely disappears at the water/kosmotrope composition where the hydrogen bonds among the water molecules in the bulk are fully optimized.

### Correlation between solvent enthalpy and partial molar heat capacity of PNiPAM in water/ethanol mixtures

**Figure 3:**
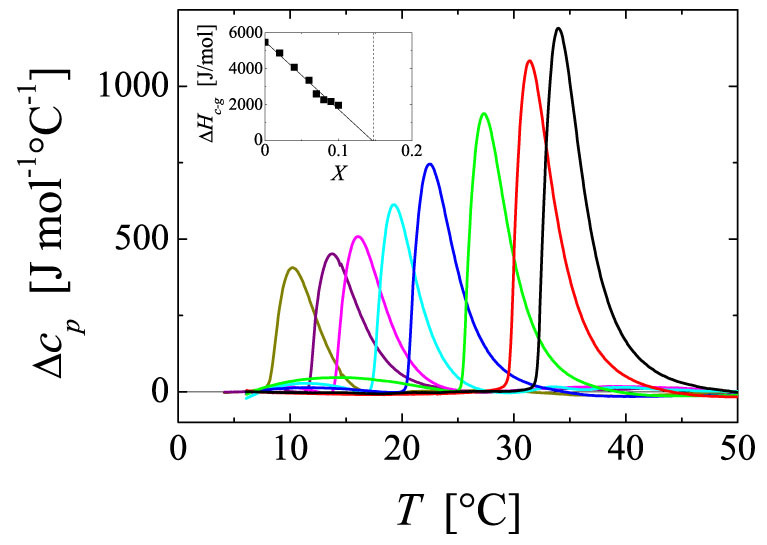
Temperature dependence of the excess partial molar heat capacity Δ*c_p_* of PNiPAM solutions in water/ethanol mixtures with varying molar fractions *X*. Solvent compositions from right to left: *X* = 0, 0.02, 0.04, 0.06, 0.07, 0.08, 0.09, 0.10. The experiments have been performed with the PNiPAM system with *M_v_* = 32 000 g/mol at a concentration of *c* = 5**·**10^−4^ g/ml. Inset: Enthalpy loss associated to the coil-to-globule transition per mol NiPAM monomer Δ*H_c-g_* (*X*) as a function of *X*. The linear extrapolation (solid line) indicates that Δ*H_c-g_* (*X*) becomes zero at X*, where both *T_c_* and Δ*H_E_* become minimal (dashed vertical line).

To further explore this concept we determine the heat effect associated to the coil-to-globule transition of PNiPAM in various water/ethanol mixtures. At this point it is worthwhile recalling that for aqueous solutions of PNiPAM there is no real distinction between the coil-to-globule transition and phase separation^24^,^27^,^28^,^31^,^32^; both phenomena coincide, such that the effect of the coil-to-globule transition can be measured at essentially any concentration. To nonetheless minimize aggregation our experiments are performed with PNiPAM solutions at fairly low concentration, *c* = 5**·**10^−4^ g/ml. In Fig. 3 we report the result obtained at a heating rate of 1°C/min in terms of excess partial molar heat capacities Δ*c_p_*, where we here refer to a mole of N-isopropyl acrylamide monomer. With increasing *X* the peak systematically shifts to lower temperatures and decreases in height and area; this clearly indicates that a decrease in the LCST is related to a decrease in the enthalpy change obtained at the coil-to-globule transition. It entails that the enthalpy of hydration of PNiPAM is becoming progressively less favourable as *X* increases. By integrating the area under the peak we obtain a quantitative estimate of Δ*H_c-g_*(*X*), the enthalpy change associated to the coil-to-globule transition of one mole of NiPAM-monomer at a given *X*. Consistent with previous experiments^27^,^57^,^58^,^59^ we find that Δ*H_c-g_* for PNiPAM in water is about 5.5 kJ/mol. This value is similar to that obtained for the hydration energy of polystyrene in water that has been recently measured by forcing the globule-to-coil transition of single polystyrene chains using atomic force microscopy^60^. This similarity between Δ*H_c-g_* measured for the amphiphilic polymer, PNiPAM, and the hydration energy of a hydrophobic polymer, such as polystyrene, further strengthens the notion that the phase behaviour of PNiPAM is primarily determined by the contributions of hydrophobic hydration, the direct interactions between water and the hydrophilic groups of PNiPAM playing a minor role for the phase transitions of this polymer. Indeed, it is worth noticing that the collapsed state of PNiPAM still contains a large amount of water^32^, which can be understood as that during the coil-to-globule transition only the water from the hydration shell surrounding the hydrophobic parts of PNiPAM is released, while water molecules interacting by direct hydrogen bonds with PNiPAM remain unaffected^61^.

**Figure 4:**
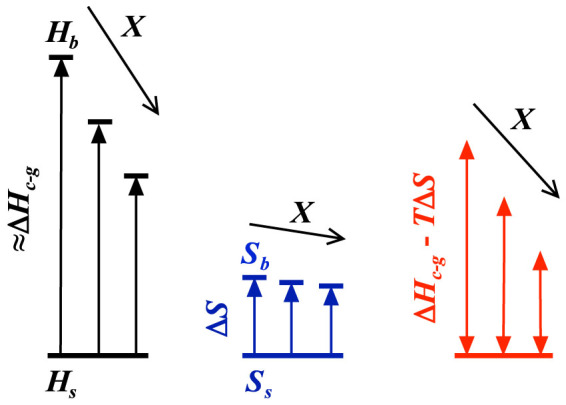
Schematic representation of the effect of the addition of alcohols on the enthalpic and entropic state of water and its implications for the critical solution temperature *T_c_* of PNiPAM. The addition of small amounts of alcohols leads to a decrease in the bulk water enthalpy *H_b_*, while the enthalpy of the water forming the hydration shell around the hydrophobic part of PNiPAM *H_s_* is not affected. As a result of this, the enthalpy difference between bulk and shell water decreases with increasing alcohol content *X*. Evidence for this is found in respectively the decrease of the excess enthalpy of mixing Δ*H_E_* with increasing *X* (Fig. 2a) and the decrease in the heat effect obtained upon release of the shell water at the coil-to-globule transition of PNiPAM Δ*H_c-g_* ≈ *H_b_* − *H_s_* with increasing *X* (inset Fig. 3). Assuming that the entropy of bulk water *S_b_* changes less dramatically with increasing *X* than *H_b_*, the difference between enthalpic and entropic contributions Δ*H_c-g_* − *T*Δ*S* is a decreasing function of *X*. The critical condition for the coil-to-globule transition or equivalently phase separation being set by Δ*H_c-g_* − *T_c_* Δ*S* ≈ 0, the lower critical solution temperature of PNiPAM is a decreasing function *X*, consistent with the behaviour observed in the low range of *X* (Fig. 1 and 2b).

Upon increasing the ethanol content Δ*H_c-g_*(*X*) systematically decreases, as shown in the inset of Fig. 3. Most remarkably, Δ*H_c-g_*(*X*) effectively extrapolates to zero at the critical composition X*, where the LCST and more importantly the excess enthalpy of mixing become minimal. This implies that when the hydrogen bonding state of bulk water becomes optimal, the decrease in enthalpy due to the hydrophobic hydration of PNiPAM becomes negligible. Qualitatively this can be understood as schematically outlined in Fig. 4. Assuming that the heat effect observed is solely due to the release of water from the hydration shell, Δ*H_c-g_* is a direct measure of the enthalpy difference between bulk and shell water, Δ*H_c-g_* ≈ *H_b_* − *H_s_*, with *H_b_* and *H_s_* the enthalpy of bulk and shell water, respectively. Regarding the critical temperature one can then define that the criterion for the limit to solubility is set by Δ*H_c-g_* ≈ *T_c_*Δ*S*, where Δ*S* = *S_b_* − *S_s_* refers to the entropy difference of bulk and shell water. The addition of alcohols leads to a decrease in *H_b_* and presumably to a lesser extent to a decrease in *S_b_*, such that Δ*H_c-g_* changes more dramatically with increasing *X* than Δ*S*. Consequently, the entropic contribution dominates at lower *T*, such that *T_c_* decreases, consistent with the observed behaviour.

This argument infers that *H_s_* and *S_s_* are not affected by the addition of a kosmotrope. Indeed, it has been shown that kosmotropes are generally excluded from the hydration shell^62^. This has led to the reasoning that preferential exclusion is at the origin of the kosmotropic effect^19^,^20^,^21^: as the free volume accessible to the kosmotrope is reduced by the exclusion from the hydration shell, there is a net gain for the entropy of the kosmotropes when the hydrophobic entities aggregate, such that the excluded volume is reduced. Such argument is generally applied to explain the kosmotropic effect on protein denaturation, where the kosmotropic effect leads to a stabilization of the native state upon both heating and cooling^21^. Indeed, the cold and heat denaturation transitions of proteins can be regarded as analogues of the globule-to-coil transitions of polymers, where the cold denaturation would then correspond to the globule-to-coil transition of PNiPAM observed at the LCST; at higher temperatures PNiPAM is in a globular, ‘folded' state, but it unfolds to a coil as it is cooled below a critical temperature. Based on our findings we argue that though preferential exclusion may explain the kosmotropic effect on heat denaturation^19^,^21^, the kosmotropic effect impacting the LCST behaviour of amphiphilic polymers or equivalently the cold denaturation of proteins is rather to be understood as that the mean energetics of water is effectively tuned by the presence of the kosmotropes. As the enthalpy of bulk water decreases upon addition of small amounts of kosmotropic solutes, so does the difference between bulk and shell water; this leads to a decrease of the LCST of amphiphilic polymers and the cold denaturation temperature of proteins and is at the origin of the kosmotropic effect in the low temperature range.

## Discussion

The correlations between solvent energetics and solution thermodynamics of PNiPAM denoted in this work effectively expose the coarse grained nature of hydrophobic hydration that is strongly determined by the mean energetics of water. Addition of small amounts of alcohols or other organic compounds classified as kosmotropic solutes lower the mean energetics of water, which is unfavourable to the hydration of hydrophobes. This is consistent with the physical considerations used in two-state models treating hydrophobic hydration as a coarse grained problem that is governed by the difference between the energy of bulk and shell water^8^,^20^,^22^,^23^.

Our findings show that the molar composition X* at which the excess enthalpy of mixing of water/kosmotrope mixtures becomes minimal is a measure of how the addition of a given kosmotrope will alter the energetic state of bulk water. For aqueous solutions of amphiphilic polymers, where hydrophobic hydration is the dominant contribution governing the phase behaviour, thermodynamic characteristics, such as the lower critical solution temperature or the heat release during the coil-to-globule transition of the polymer, evolve relative to X*. This molar composition can effectively be regarded as the upper limit at which the presence of a kosmotropic solute solely decreases the energetics of water due to the kosmotropic effect. Note that the considerations exposed in this work are limited to kosmotropes that are neutral organic solutes, which are often considered as co-solvents. Below X*, however, we can simply think of the solvent mixture as an aqueous medium with an energetic state that is set by the amount and type of kosmotrope added, the overall characteristics of hydrophobic hydration remaining the same as in pure water. Above this limit the solvation properties of the kosmotrope as a co-solvent become relevant and the mechanism of hydrophobic hydration does not operate anymore. Such notion provides a simple guideline for designing experiments exploring and/or exploiting the tunability of amphiphilic assemblies in water/kosmotrope mixtures; a guideline that does not require the full description of the thermodynamics, which may be rather complex in such multi-component systems.

There is certainly no doubt that the detailed configuration of the backbone and side groups of an amphiphilic polymer determine the relative importance of the different contributions entering the solution thermodynamics of such polymers. However, the entropic contributions owing to the solvation of polymers are generally low as compared to those of small molecules. The likelihood that hydrophobic hydration becomes the predominant contribution governing the phase behaviour of an amphiphilic polymer is thus rather high. This is the case for PNiPAM and it entails that the critical transition temperatures are independent of the actual polymeric contributions that depend on the polymer molecular weight and architecture and the polymer concentration; this is because hydrophobic hydration is a solvent state problem, governed by the energy difference between bulk and shell water, which is independent of the polymer. Testing for such independence should be a valuable means to evaluate the contribution of hydrophobic hydration to the thermodynamics of solutions of any amphiphilic polymer. The prevalence of hydrophobic hydration in governing the phase behaviour of PNiPAM further points to the use of PNiPAM as a probe for the mean energetics of water, which can be exploited in studies of the water state and in particular to its modulation via the kosmotropic effect induced by the addition of small amounts of neutral organic solutes.

## Methods

### Samples

We use three PNiPAM systems from Polymer Source Inc. The characteristics of these systems differ in their viscosity averaged molecular weights *M_v_* and polydispersities *M_w_*/*M_n_*, where *M_w_* and *M_n_* respectively denote the weight and number averaged molecular weight. In the series of experiments probing the molecular weight and concentration dependence of the LCST we use PNiPAM systems with respectively *M_v_* = 39 000 g/mol − *M_w_/M_n_* = 1.45 and *M_v_* = 465 500 g/mol − *M_w_/M_n_* = 4.9. In the series of experiments probing the calorimetric properties of the PNiPAM solutions the characteristics of the PNiPAM system are: *M_v_* = 32 000 g/mol − *M_w_/M_n_* = 1.5. The PNiPAM solutions at various alcohol molar fractions *X* are prepared by mixing stock solutions of PNiPAM of a given molecular weight and concentration in pure water (Milli-Q) and pure analytical grade alcohol, so to obtain the desired alcohol molar fraction *X*. As alcohols we use methanol, ethanol, iso-propanol and propanol.

### Determination of critical solution temperature

The critical solution temperatures *T_c_* are determined in cloud point measurements. For these measurements we place sealed glass tubes, containing the aqueous PNiPAM solutions, in a homemade temperature cell, where the temperature can be controlled in a temperature range of −20°C to 60°C with a precision of ±0.1°C. After a first approximate assessment of *T_c_* using a fast temperature ramp we approach the critical temperatures from below or above depending on whether the transition is characterized by a lower or upper critical solution temperature in steps of 0.1°C, where we allow the solutions to equilibrate for at least 5 minutes at each temperature. The cloud point is determined by visually assessing the onset to turbidity. The critical solution temperatures obtained in control experiments using a commercial light scattering apparatus to measure the cloud point as the onset to larger scattering intensity are consistent with those determined visually.

### Calorimetric measurements

The heat effect associated to the coil-to-globule transition of PNiPAM in water/ethanol mixtures is measured using a commercial differential scanning micro-calorimeter (Microcal VP-DSC). In these measurements the difference in the heat capacity of solutions containing PNiPAM with *M_v_* = 32 000 g/mol at a concentration of *c* = 5**·**10^−4^ g/ml and the heat capacity of the solvent are measured upon heating at a rate of 1°C/min. Tests at lower scanning rates yield identical results, indicating that the chosen heating rate is low enough to guarantee that the system remains thermally equilibrated during the measurement.

## Author Contributions

I.B., D.C.E.C. and V.T. designed research; I.B. and D.C.E.C. performed research; I.B., D.C.E.C., I.J., P.D.L.R. and V.T. analysed data; and I.B., D.C.E.C. and V.T. wrote the paper.

## Figures and Tables

**Table 1:** Correlations between solvent and PNiPAM solution thermodynamics. Solvent compositions at which the LCST of PNiPAM solutions becomes minimal X*(*T_c_*) in comparison to those at which the excess enthalpy of mixing of the solvent mixture becomes minimal X*(Δ*H_E_*). For all water/kosmotrope mixtures X*(*T_c_*) ≈ X*(Δ*H_E_*), while X*(*T_c_*) and X*(Δ*H_E_*) are uncorrelated for the water/chaotrope mixtures marked in *italic*. The data relating to Δ*H_E_*(X*) are obtained at X*. Note that Δ*H_E_*(X*) is here reported for 25°C; it will vary with temperature, while X*(Δ*H_E_*) is temperature independent^35^,^36^,^37^,^38^,^41^

	X*(*T_c_*)	X*(Δ*H_E_*)	Δ*H_E_*(X*) [J/mol]
Methanol	0.31 ± 0.01	0.32 ± 0.01^35^	−854 ± 10^35^
Ethanol	0.16 ± 0.01	0.15 ± 0.01^35^	−753 ± 10^35^
Iso-Propanol	0.10 ± 0.01	0.10 ± 0.01^35^	−654 ± 10^35^
Propanol	0.08 ± 0.01	0.07 ± 0.01^35^	−363 ± 10^35^
Acetone	0.14 ± 0.01^26^	0.14 ± 0.01^39^	−672 ± 10^39^
Dioxane	0.11 ± 0.01^29^	0.13 ± 0.01^40^	−557 ± 10^40^
THF	0.15 ± 0.01^29^	0.15 ± 0.01^41^	−734 ± 10^41^
*DMSO*	*0.18* ± *0.01*^26^	*0.35* ± *0.01*^42^	−*2844* ± *10*^42^
*DMF*	*0.27* ± *0.01*^26^	*0.35* ± *0.01*^42^	−*2152* ± *10*^42^
